# Supplementary material for: Prostate cancer histopathology using label-free multispectral deep-UV microscopy quantifies phenotypes of tumor aggressiveness and enables multiple diagnostic virtual stains
Source: Sci Rep. 2022 Jun 4;12:9329. doi: 10.1038/s41598-022-13332-9 (PMC9167293; doi:10.1038/s41598-022-13332-9)
Supplement: Supplementary file 1 — Supplementary Information. [file 41598_2022_13332_MOESM1_ESM.pdf]

Supplementary Materials for

Prostate cancer histopathology using label-free multispectral deep-UV microscopy quantifies phenotypes of tumor aggressiveness and enables multiple diagnostic virtual stains

Soheil Soltani, Ashkan Ojaghi, Hui Qiao, Nischita Kaza, Xinyang Li, Qionghai Dai, Adeboye O Osunkoya, Francisco E Robles*

*Corresponding author Email: robles@gatech.edu

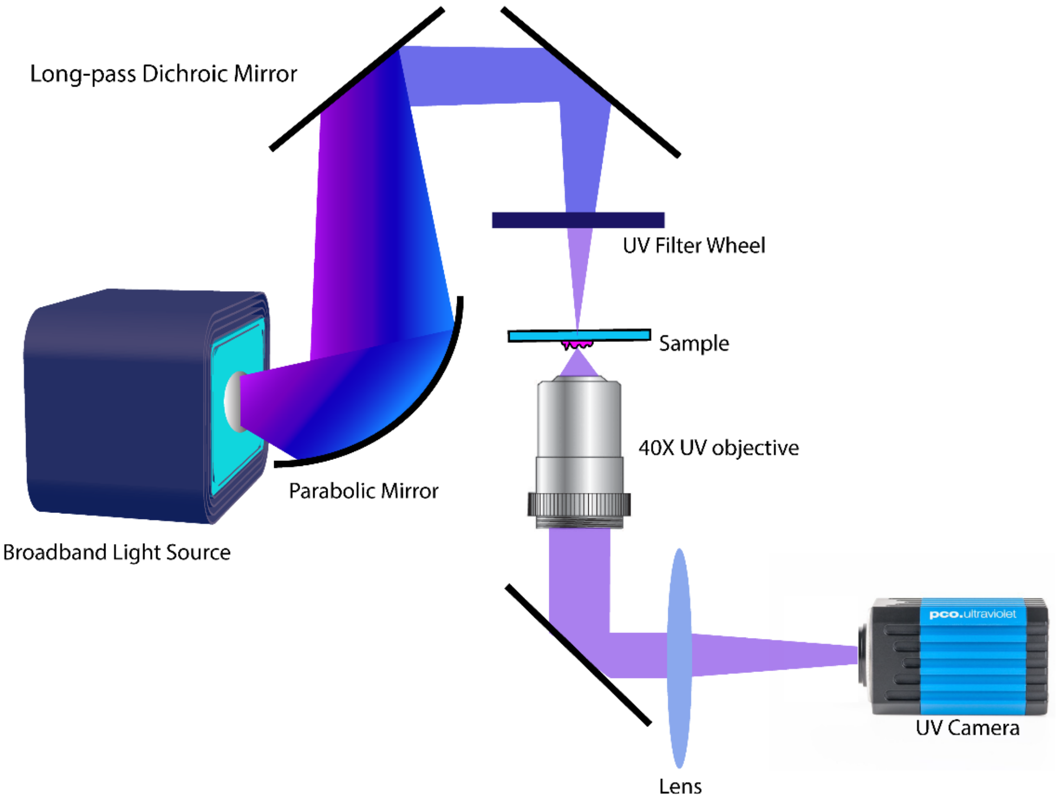

Fig. S1. A schematic of the multi-spectral deep UV microscope. The source provides a broadband output beam (~200 nm to 2000 nm) that is focused on the sample using a parabolic mirror. A dichroic mirror is used to only select deep UV region of the spectrum (200-550 nm). The transmitted light is collected using a 40X UV objective and is relayed on the camera using a biconvex lens

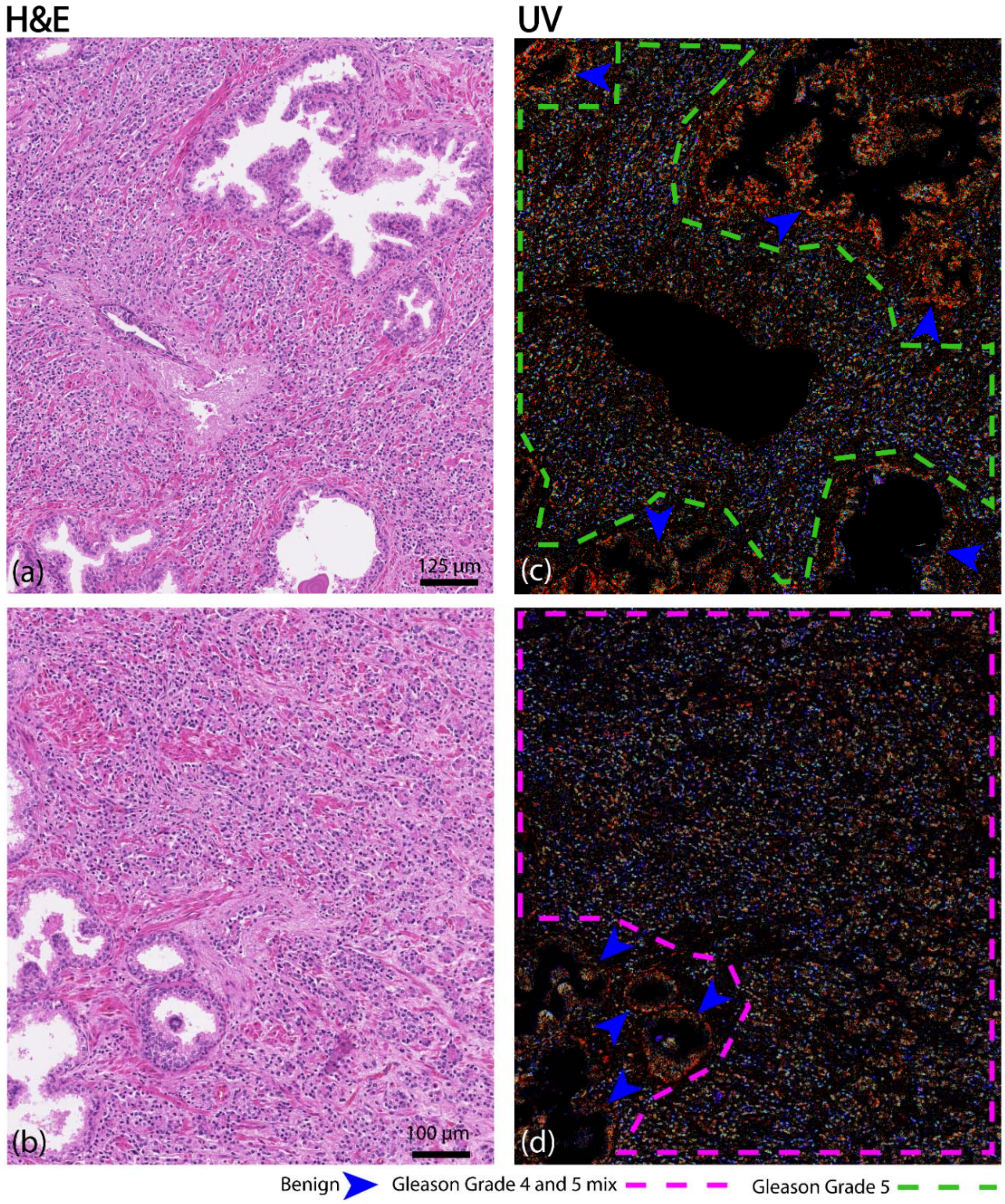

Fig. S2. Two examples of prostate cancer regions with aggressive forms of cancer (Gleason Grade 5). It is important to mention that the region in (b) starts from foci of Gleason Grade 4 in the right side of the image and gradually cancer regions with Gleason Grade 5 appear in the top left side of the region (there are not pure Gleason Grade 4 or Gleason Grade 5 regions). Some levels of inflammation are also present.

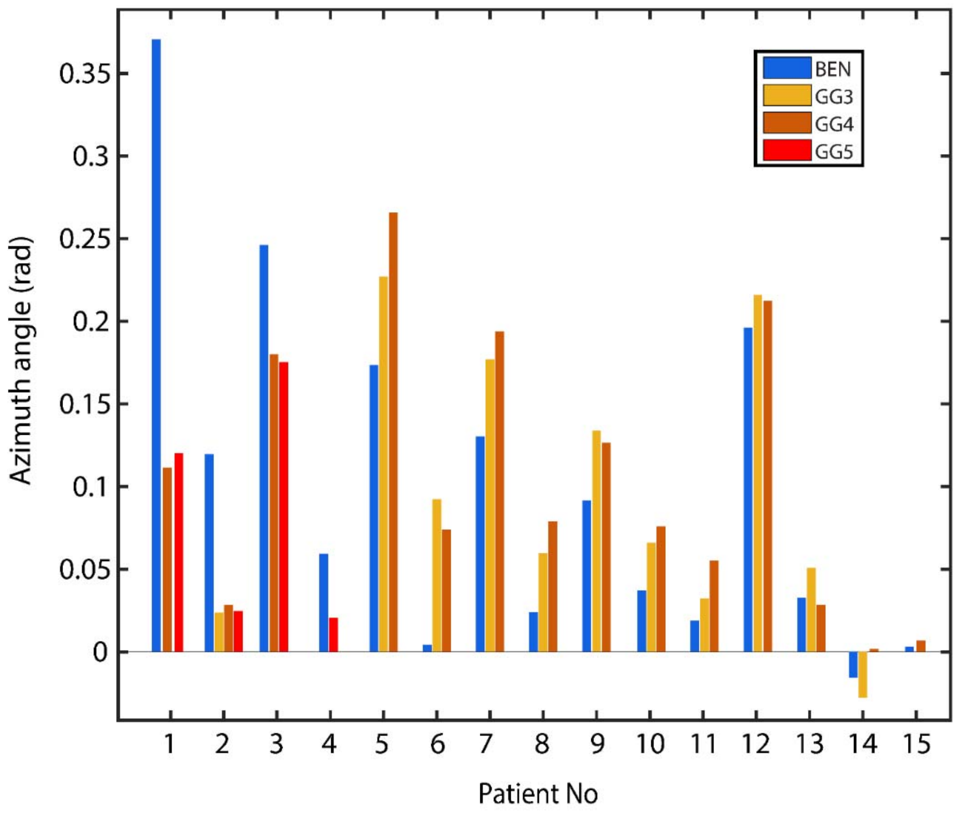

Fig. S3. **Bar plot of the absolute azimuth angle CoM.**
